# Anti-high-mobility group box-1 (HMGB1) mediates the apoptosis of alveolar epithelial cells (AEC) by receptor of advanced glycation end-products (RAGE)/c-Jun N-terminal kinase (JNK) pathway in the rats of crush injuries

**DOI:** 10.1186/s13018-021-02903-7

**Published:** 2022-01-15

**Authors:** Bin-Fei Zhang, Wei Song, Jun Wang, Peng-Fei Wen, Yu-Min Zhang

**Affiliations:** Department of Joint Surgery, Honghui Hospital, Xi’an Jiaotong University, No. 555 Youyi East Road, Beilin District, Xi’an, Shaanxi Province 710054 People’s Republic of China

**Keywords:** Crush injury, High-mobility group box-1, Receptor of advanced glycation end-products (RAGE), c-Jun N-terminal kinase (JNK), SP600125, Apoptosis, Alveolar epithelial cells

## Abstract

**Objectives:**

The lung injury is often secondary to severe trauma. In the model of crush syndrome, there may be secondary lung injury. We hypothesize that high-mobility group box 1 (HMGB1), released from muscle tissue, mediates the apoptosis of alveolar epithelial cells (AEC) via HMGB1/Receptor of advanced glycation end-products (RAGE)/c-Jun N-terminal kinase (JNK) pathway. The study aimed to investigate how HMGB1 mediated the apoptosis of AEC in the rat model.

**Methods:**

Seventy-five SD male rats were randomly divided into five groups: CS, CS + vehicle, CS + Ethyl pyruvate (EP), CS + FPS-ZM1 group, and CS + SP600125 groups. When the rats CS model were completed after 24 h, the rats were sacrificed. We collected the serum and the whole lung tissues. Inflammatory cytokines were measured in serum samples. Western blot and RT-qPCR were used to quantify the protein and mRNA. Lastly, apoptotic cells were detected by TUNEL. We used SPSS 25.0 for statistical analyses.

**Results:**

Nine rats died during the experiments. Dead rats were excluded from further analysis. Compared to the CS group, levels of HMGB1 and inflammatory cytokines in serum were downregulated in CS + EP, CS + FPS-ZM1, and CS + SP600125 groups. Western blot and RT-qPCR analysis revealed a significant downregulation of HMGB1, RAGE, and phosphorylated-JNK in CS + EP, CS + FPS-ZM1, and CS + SP600125 groups, compared with the CS groups, excluding total-JNK mRNA. Apoptosis of AEC was used TUNEL to assess. We found the TUNEL-positive cells were downregulated in CS + EP, CS + FPS-ZM1, and CS + SP600125 groups.

**Conclusion:**

The remote lung injury begins early after crush injuries. The HMGB1/RAGE/JNK signaling axis is an attractive target to abrogate the apoptosis of AEC after crush injuries.

## Introduction

Crush injuries are severe soft tissues damage in the field of orthopedic trauma. Prolonged compression of limb muscles and subsequent decompression is essential in the development of crush syndrome (CS) [1], including hyperkalemia, metabolic acidosis, hypovolemic shock, acute kidney injury, and disseminated intravascular coagulation [2]. The sequential damage in the kidney after crush injury is not the unique dysfunctional organ [3]. It has been proved that the secondary damage in the lung [4–6], heart [7], and liver [8] contributes to multiple organs failure [9]. In fact, the pathophysiology following CS is a procedure of systemic inflammatory introducing injuries.

The lung injury has been observed clinically after severe trauma. This lung injury is secondary to the trauma, and likely due to an elevation in leukocytes and inflammatory cytokines in the lung tissue [10]. Duration of the treatment of trauma and the lung injury is often paid attention to when finding worse pulmonary function. It may be that the lung injury begins at the time of decompression. Therefore, we should notice that the remote lung injury at the early stage.

Currently, numerous studies have focused on a proinflammatory mediator, high-mobility group box 1 (HMGB1) [11–13]. In the model of CS, the levels of HMGB1 in muscle increased to peak at 12 h after decompression in the mice model [14]. In addition, the administration of anti-HMGB1 antibody improves survival rate and suppresses serum levels of HMGB1 and inflammation cytokines [6]. Significantly, the treatment for HMGB1 could ameliorate lung injury [15, 16].

However, the relationship between extracellular HMGB1 and lung injury is uncertain in crush injuries. The previous study has shown that HMGB1 is released from muscle tissue in response to damage immediately, and the level of serum HMGB1 increases quickly after decompression in the rats model [6]. The HMGB1 can be passively released from damaged cells as a proinflammatory alarmin [17]. When released HMGB1 enters the systemic circulation, the sequential response in remote lung injury follows. The receptor of advanced glycation end-products (RAGE) is a cell surface transmembrane multiligand receptor, and it can bind a large variety of endogenous ligands, such as HMGB1, leading to its classification as a pattern recognition receptor [18]. In the LPS-induced lung injury, the injury was found via HMGB1-RAGE signaling in alveolar epithelial cells (AEC). In addition, c-Jun N-terminal kinase (JNK) is an essential transducing enzyme that is involved in apoptosis of AEC [19], and JNK inhibitor SP600125 could improve cell apoptosis in acute lung injury [20]. The RAGE/JNK pathway has also been proved in participating the inflammation response [21].

Thus, we hypothesize that HMGB1, released from muscle tissue, mediates the apoptosis of AEC via the HMGB1/RAGE/JNK pathway. This study aimed to investigate the hypothesis via different inhibitors in the rats model.

## Materials and methods

### Animals

Seventy-five SD male rats weighing 250–300 g were purchased from the Laboratory Animal Center of Xi’an Jiaotong University. All animal procedures followed the ARRIVE guidelines [22] and the National Institutes of Health guide for the care and use of laboratory animals [23]. The Ethics Committee approved the study of Honghui Hospital, Xi’an Jiaotong University (2018025).

### Treatment

The rats were housed and fed in a temperature of 22 ± 1 °C and humidity 45–55% environment with a standardized light–dark cycle (12 h day/night), and they were free to eat and drink. After one week of adaptive feeding, they were randomly divided into five groups: CS group, CS + vehicle group, CS + Ethyl pyruvate (EP) group, CS + FPS-ZM1 group, and CS + SP600125 group. The concentration of ethyl pyruvate, FPS-ZM1, and SP600125 was 50 mg/kg, 5 mg/kg, and 15 mg/kg, respectively.

We injected the above drugs into the intraperitoneal cavity before compression 0.5 h and used DMSO as the vehicle. (The injection volume was 0.7 mL.) CS + vehicle group was injected with the same volume of DMSO as a negative control, and the CS group was used as a blank control.

### Rats model of CS

After administering inhibitors 0.5 h, the SD rats were anesthetized by intraperitoneal injection of 3% sodium pentobarbital (30 mg/kg). After anesthesia, the rats were fixed in the prone position, and the hind limbs were compressed at 4.5 cm away from the ankles, with 20 kg [24] heavy for 3 h. Because the mortality was high when compressing 6 h in a preliminary study, we chose to compress 3 h. The body temperature of the rats was maintained at 37 ± 0.5 °C during this period. After the compression was removed, the rats were allowed to recover for 24 h. When the recovery time was up to 24 h, the blood of rats was collected, the serum was separated, and then the rats were sacrificed. We collected the whole lung tissue and divided each lung tissue into two parts: one part of the lung tissue was stored in liquid nitrogen, and the other part of the lung tissue was fixed with 4% paraformaldehyde solution for later use.

### ELISA

All serum samples were tested for HMGB1 (FineTest, Wuhan, China), tumor necrosis factor-α (TNF-α) (Wanleibio, Shenyang, China), IL-1β (Wanleibio, Shenyang, China), and IL-6 (Wanleibio, Shenyang, China), using a rat ELISA kit. The ELISA assay was performed following the manufacturer's protocols. The optical density of each well was measured spectrophotometrically at 450 nm within 5 min after adding the stop solution using a microplate reader (ELX-800, BIOTEK, USA).

### Western blot

The frozen tissue samples were solubilized in RIPA buffer on ice using a homogenizer. Samples (50 μg/lane) were separated on a sodium dodecyl sulfate–polyacrylamide gel 12% gel) and electrotransferred onto polyvinylidene fluoride membranes. After 2 h incubation in blocking solution (5% non-fat milk in 20 mM Tris–HCl, 150 mM NaCl, 0.1% Tween-20; TBST), the membranes were blotted with primary antibodies against HMGB1 (1:1000, A19529, ABclonal), RAGE (1:1000, AF5309, Affinity), p-JNK (1:500, WL01295, Wanleibio), JNK (1:500, WL01813, Wanleibio), or β-actin (1:500, Wanleibio) overnight at 4 °C. After extensive rinsing with TBST buffer, the blots were incubated with HRP-conjugated secondary antibodies (Abgent, San Diego, CA, USA). The density of the bands was analyzed by Quantity One software version 4.62 (Bio-Rad, USA).

### Real-time qPCR

Total RNA from the rats’ lung tissues was extracted by TRIpure reagent (RP1001, BioTeke, China). A commercial reverse transcription system BeyoRT II M-MLV (D7160L, Beyotime Biotechnology, China) was used to synthesize cDNA from purified total RNA. qRT-PCR was conducted using SYBR Green (SY1020, Solarbio, China) on a thermocycler (Exicycler™ 96, BIONEER, Korea). The sequences of primers used are listed in Table 1. β-actin was used as an internal control for indicated genes. The relative expression of a target gene was determined by the 2^−△△CT^ method (NANO 2000, Thermo, USA).

**Table 1:** Sequences of primers (5′–3′)

Primer	Sequence	Primer length	Tm	Product length
HMGB1 F	TGACAAGGCTCGTTATGAAAG	21	55.8	186
HMGB1 R	TTCTTCGCAACATCACCAAT	20	56.2	
RAGE F	GACGGGACTCTTCACGCTTCG	21	64.4	192
RAGE R	CCACCTTCAGGCTCAACCAAC	21	61.8	
JNK F	GATTTGGAGGAGCGAACTAA	20	54.7	161
JNK R	CTGCTGTCTGTATCCGAGGC	20	58	
β-actin F	GGAGATTACTGCCCTGGCTCCTAGC	25	60.1	155
β-actin R	GGCCGGACTCATCGTACTCCTGCTT	25	62	

### TUNEL staining

We used a terminal deoxynucleotidyl transferase-mediated dUTP nick end-labeling (TUNEL) reaction utilizing TUNEL Kit (WLA127a, Wanleibio, China) to detect apoptotic cells. The tissue sections were deparaffinized with xylene and treated with H2O2 to inactivate endogenous peroxidase. After washing with PBS, the following operations were completed in a dark room. The specimens were incubated in the red labeling reaction mixture containing terminal deoxynucleotidyl transferase. The sections were then counterstained with a blue DAPI solution (D106471, Aladdin-e, China). Finally, the fluorescence images were collected in a camera system (DP73, OLYMPUS, Japan).

### Statistical analysis

SPSS 25.0 (SPSS Inc., Chicago, IL, USA) was used for statistical analyses. All data were presented as mean ± SD. Comparisons among multiple groups were performed using ANOVA. Comparisons between the two groups were performed using the LSD test. *P* < 0.05 was considered statistically significant.

## Results

### Mortality rate and general observations

In total, nine rats died after the compression and administration of drugs during the experiments. The number of deaths was 2, 3, 1, 2, and 1 in CS, CS + vehicle, CS + EP, CS + FPS-ZM1, and CS + SP600125 groups, respectively. There was no significant difference in mortality rate (*P* = 0.778). Dead rats were excluded from further analysis. After the modeling, activities of rats reduced obviously, with both hind limbs prolapsing. In all living rats, we found injured skeletal muscles to be hemorrhagic and with edema.

### The levels of HMGB1 and inflammatory cytokines in serum

**Fig. 1:**
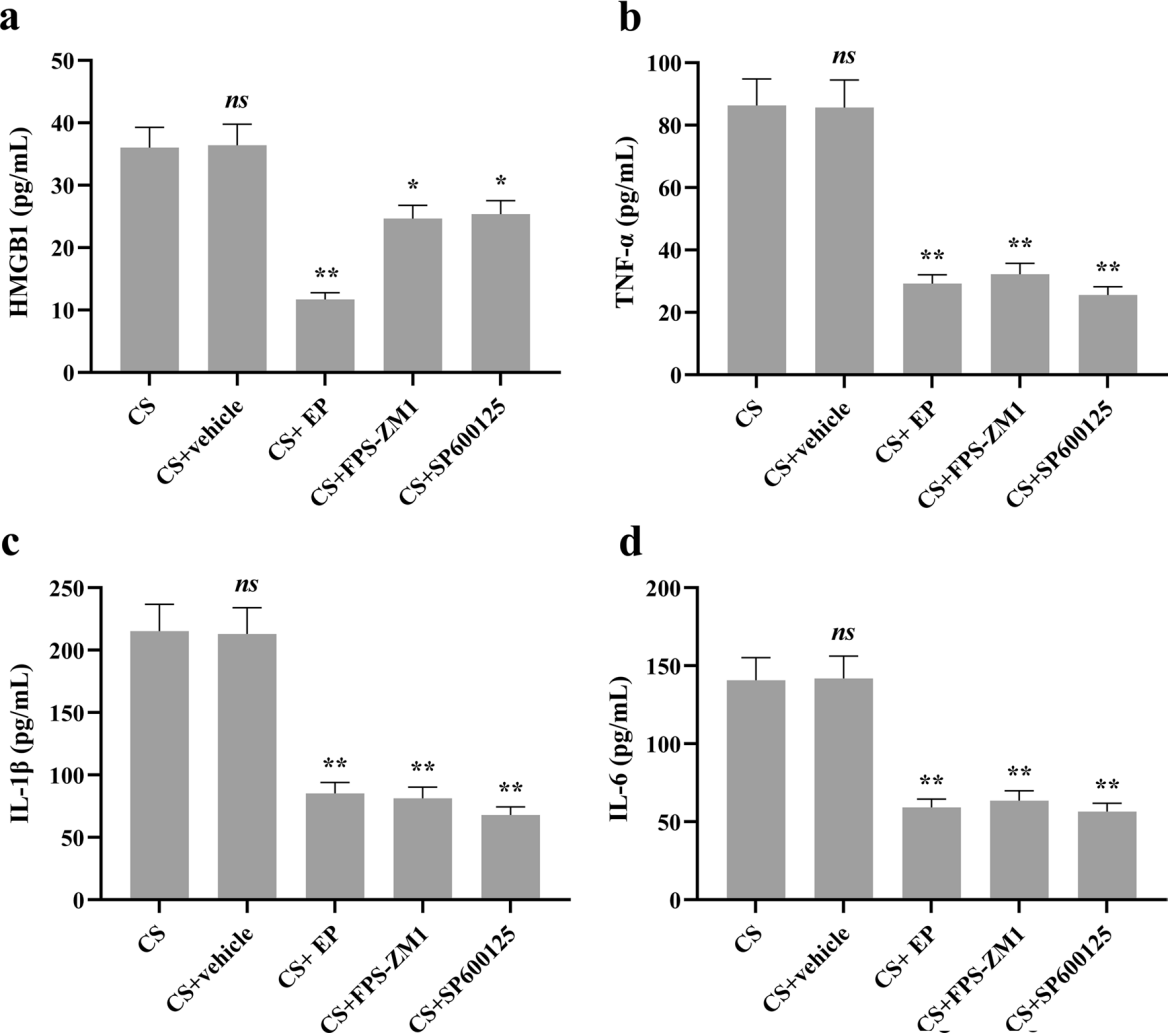
EP, FPS-ZM1, and SP600125 affect the expression of HMGB1 and inflammatory cytokines in serum. Relative levels of HMGB1, TNF-α, IL-1β, and IL-6 were analyzed using ELISA kits. The HMGB1 and all inflammatory cytokines were lower in the later three pretreatment groups. Results were expressed as means ± SDs. The number of rats were 13, 12, 14, 13, and 14 in CS, CS + vehicle, CS + EP, CS + FPS-ZM1, and CS + SP600125 groups, respectively. Statistical significance: *ns P* ≥ 0.05, *P < 0.05 and **P < 0.01, versus CS group

When testing the levels of serum HMGB1, we found that there was no significant statistical difference between the CS group and CS + vehicle group. Compared to the CS group, the level of HMGB1 was downregulated in CS + EP, CS + FPS-ZM1, and CS + SP600125 groups (Fig. 1a).

As for the inflammatory cytokines of TNF-α, IL-1β, and IL-6, there was no significant difference between the CS and CS + vehicle groups. However, the levels of these cytokines were lower in CS + EP, CS + FPS-ZM1, and CS + SP600125 groups, compared to the CS group (Fig. 1b–d).

### Effect of EP, FPS-ZM1, and SP600125 on the protein expression of HMGB1, RAGE, and JNK

**Fig. 2:**
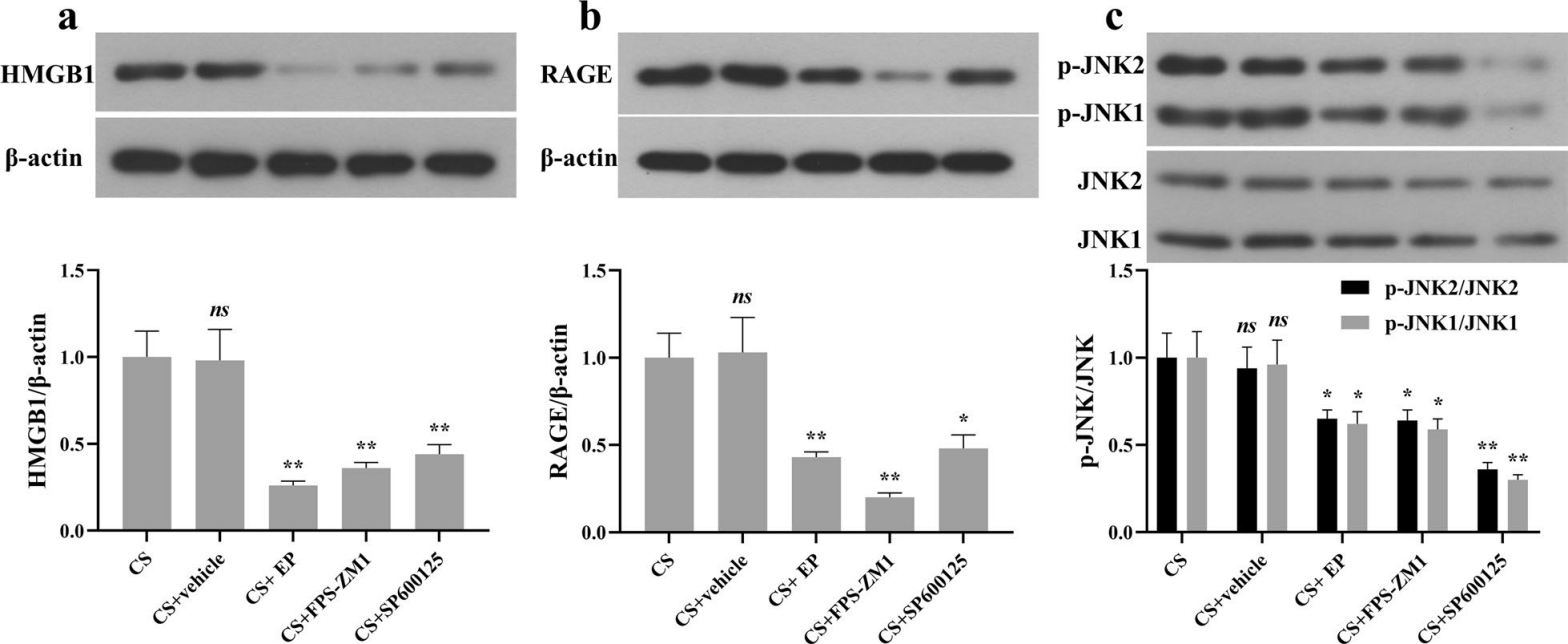
EP, FPS-ZM1, and SP600125 affect the expression of HMGB1, RAGE, and JNK in lung tissue. Relative levels of HMGB1, RAGE, and JNK were analyzed using Western blot. The HMGB1, RAGE, and p-JNK were lower in the later three pretreatment groups. Results were expressed as means ± SDs. The number of rats were 13, 12, 14, 13, and 14 in CS, CS + vehicle, CS + EP, CS + FPS-ZM1, and CS + SP600125 groups, respectively. Statistical significance: *ns P* ≥ 0.05, *P < 0.05 and **P < 0.01, versus CS group

Western blot analysis revealed a significant downregulation of HMGB1 in CS + EP, CS + FPS-ZM1, and CS + SP600125 groups, compared with the CS groups (Fig. 2a). As for the RAGE and p-JNK, there was also significant downregulation in CS + EP, CS + FPS-ZM1, and CS + SP600125 groups (Fig. 2b, c).

### Effect of EP, FPS-ZM1, and SP600125 on the mRNA expression of HMGB1, RAGE, and JNK

**Fig. 3:**
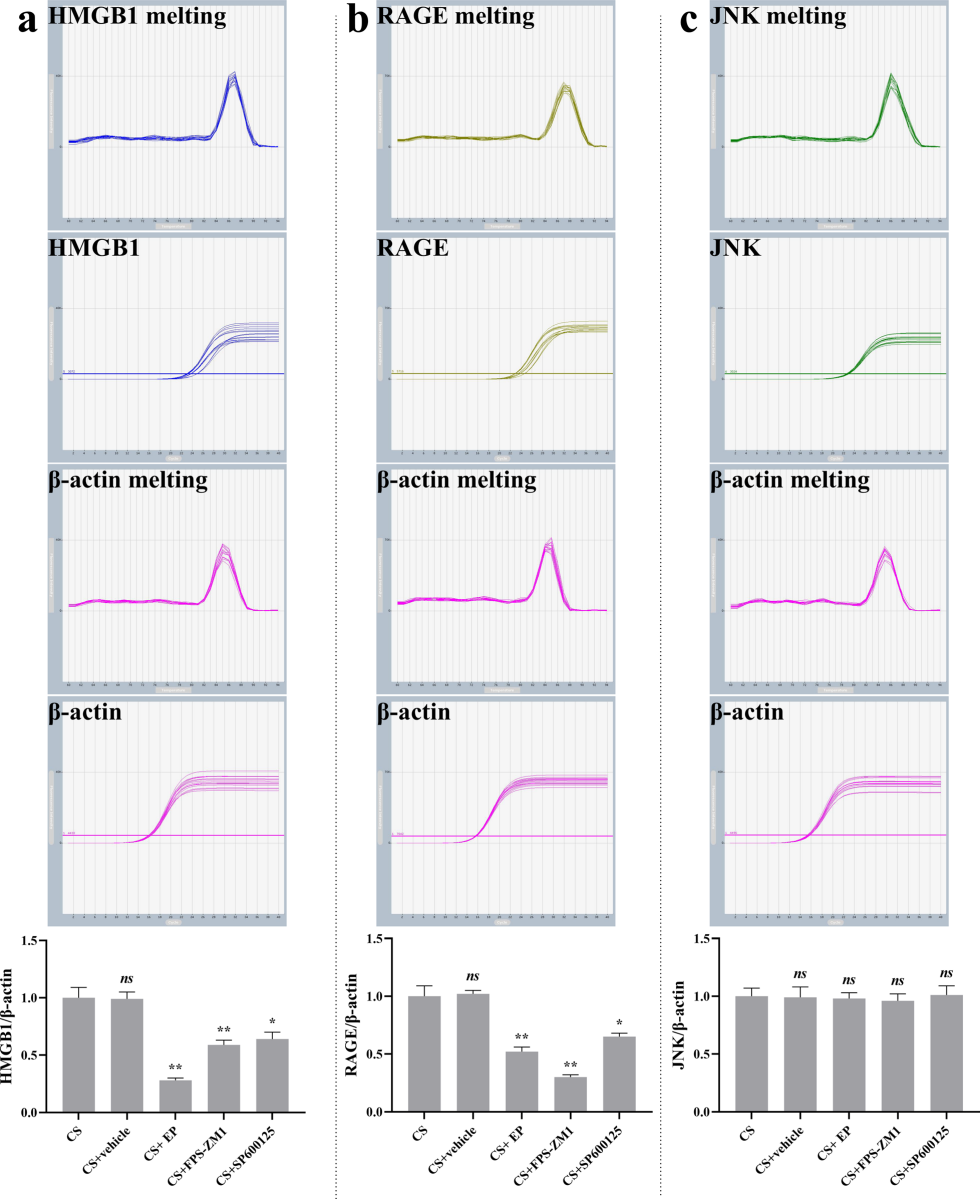
EP, FPS-ZM1, and SP600125 affect the mRNA expression of HMGB1, RAGE, and JNK in lung tissue. Relative levels of HMGB1, RAGE, and JNK were analyzed using RT-qPCR. The HMGB1, RAGE, and p-JNK were lower in the later three pretreatment groups. Results were expressed as means ± SDs. The number of rats were 13, 12, 14, 13, and 14 in CS, CS + vehicle, CS + EP, CS + FPS-ZM1, and CS + SP600125 groups, respectively. Statistical significance: *ns P* ≥ 0.05, *P < 0.05 and **P < 0.01, versus CS group

RT-qPCR analysis revealed a significant downregulation of HMGB1 and RAGE mRNA in CS + EP, CS + FPS-ZM1, and CS + SP600125 groups, compared with the CS groups (Fig. 3a, b). As for the mRNA expression of JNK, there was no difference in the five groups (Fig. 3c).

### Apoptosis of AEC

**Fig. 4:**
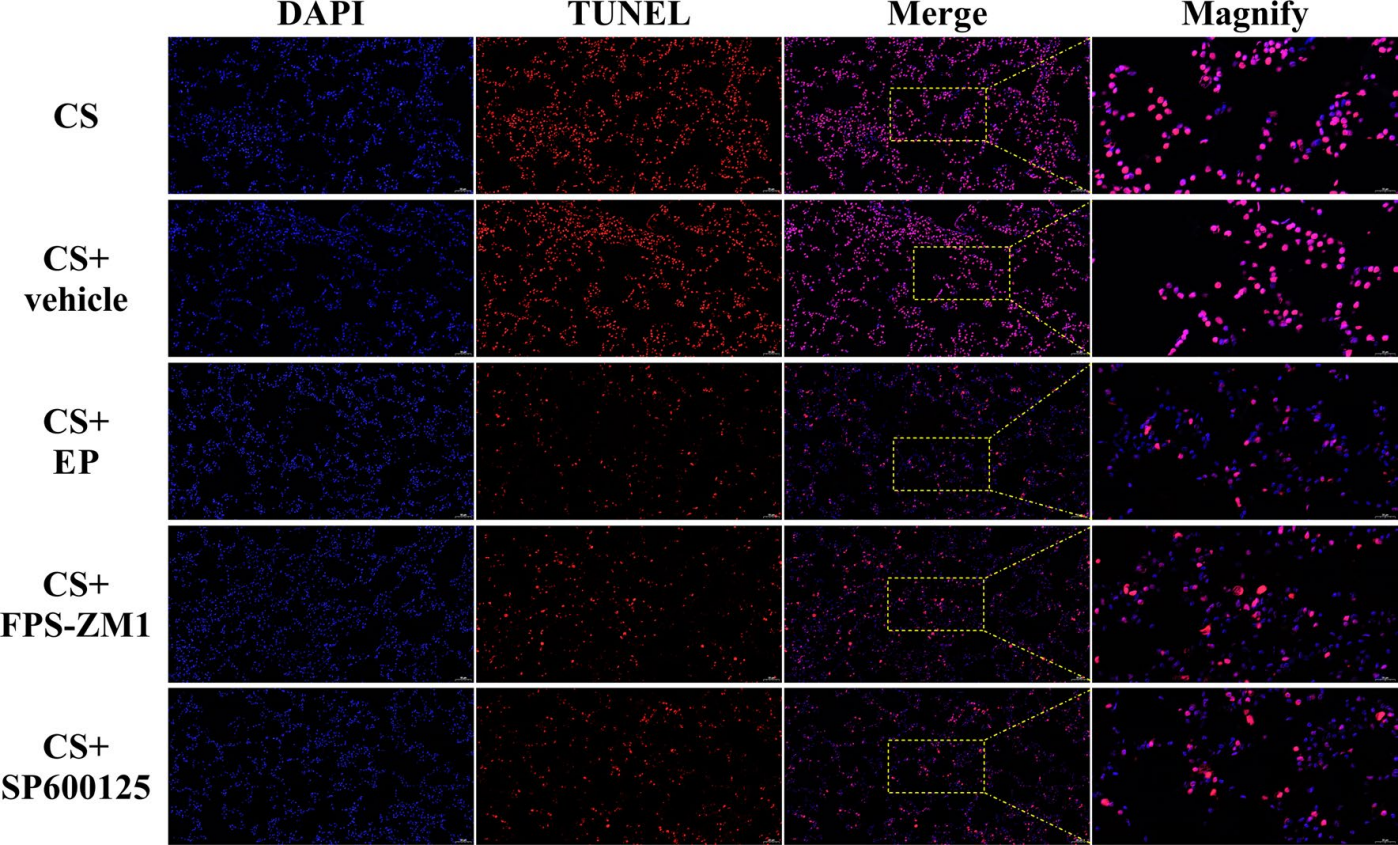
EP, FPS-ZM1, and SP600125 affect TUNEL-positive cells in lung tissue. The TUNEL-positive cells in CS + EP, CS + FPS-ZM1, and CS + SP600125 groups were lower than the CS group. The number of rats were 13, 12, 14, 13, and 14 in CS, CS + vehicle, CS + EP, CS + FPS-ZM1, and CS + SP600125 groups, respectively. We collected five slices in every rat and randomly selected five magnified visions in every slice

We used TUNEL to assess the apoptosis of AEC. As shown in Fig. 4, most of the AEC were TUNEL-positive cells in CS and CS + vehicle groups, and the number of positive cells decreased in the other three groups. After the statistic, we found there was no significant difference between the CS and CS + vehicle groups (*P* > 0.05) and TUNEL-positive cells in CS + EP, CS + FPS-ZM1, and CS + SP600125 groups were lower than the CS group (*P* < 0.05).

## Discussion

In clinical, the lung injury maybe start at the early stage after crush injuries. The surgeons should notice the dynamic changes in pulmonary function. There were two main reasons we designed this study. On the one hand, some animals had the amount of pink foam in the mouth and nose when making animal models. We highly suspect the occurrence of secondary lung injury in the crush injury. On the other hand, this signaling pathway has not been verified in CS animal models. Thus, we conducted this animal experiment. In this study, we explored the potential role of the HMGB1/RAGE/JNK pathway on apoptosis of AEC to show the damage to lung tissue under the model of crush injuries. Our results indicate the following main findings. Firstly, the HMGB1 in muscle is rapidly released to the systemic circulation, and the remote lung injury begins early after crush injuries. Secondly, the HMGB1/RAGE/JNK pathway is involved in the pathophysiology of lung injury following CS. Thirdly, inhibitors of HMGB1, RAGE, and JNK could alleviate the apoptosis of AEC and serum inflammatory cytokines.

In terms of animal modeling, we chose SD rats and compression for 3 h. There are two reasons. The first reason was the degree of muscle damage at 3 h was the same as those at 6 h in the preliminary experiment, but there were more necrotic cells at 6 h. The second reason was that the mortality rate of rats at 6 h was too high, nearly 70–80%. It meant requiring too many experimental animals. In particular, the survival animals may have the possibility of selection bias, which would affect the reliability of the experimental results.

As for the HMGB1, RAGE, and JNK signaling inhibitors, we have chosen EP, FPS-ZM1, and SP600125 for intervention. The role of EP was chelating Ca^2+^ directly and responsible for the suppression of HMGB1 [25]. EP has been used to abrogate the pathological effects of HMGB1 [25, 26]. FPS-ZM1 is a blood–brain-barrier permeant, tertiary amide compound which is a high-affinity RAGE-specific inhibitor, blocking Aβ binding to the V domain of RAGE [27]. FPS-ZM1 was often used to inhibit the role of RAGE in an animal experiment [28, 29]. Moreover, an inhibitor of JNK, SP600125 [20], has been widely used. Thus, these administrations were specific blocking drugs to the signaling axis. We chose to give the drugs into the intraperitoneal cavity before compression to ensure they completely play the role.

In terms of detection methods, we adopted western blot and RT-qPCR techniques for mechanism exploration. Since the protein and mRNA expression trends were similar, the experimental results were relatively reliable. In addition, TUNEL was used to detect apoptosis of AEC [30]. Thus, these methods were typical and reliable.

The HMGB1/RAGE/JNK pathway has been studied in some studies, and the role of this pathway has been established in dopaminergic neurons [31], degenerated human nucleus pulposus cells [32]. In this study, we have identified the signaling axis of HMGB1/RAGE/JNK in lung injury after CS. The HMGB1 in muscle was released to the systemic circulation. When HMGB1 arrived at the lung, the RAGE/JNK pathway in AEC was activated, and the pathophysiology of lung injury began. Lastly, apoptosis becomes one of the damaged types of AEC. Furthermore, the treatment of inhibiting or blocking the role of HMGB1 may moderate the lung injury.

However, there are some limitations to this study. Firstly, we could not confirm whether inflammatory cytokines are released from damaged muscle cells or a response from other cells. Secondly, it is uncertain whether the apoptotic cell type is AEC type I or II or both. The above shortcomings need to be further implemented in cell research.

## Conclusions

The remote lung injury begins early after crush injuries. The HMGB1/RAGE/JNK signaling axis is an attractive target to abrogate the apoptosis of AEC after crush injuries.

## Data Availability

Please contact author for data requests.

## Abbreviations

CS: Crush syndrome
HMGB1: High-mobility group box-1 protein
RAGE: Receptor of advanced glycation end-products
JNK: C-Jun N-terminal kinase
AEC: Alveolar epithelial cells
TNF-α: Tumor necrosis factor-α
TUNEL: Terminal deoxynucleotidyl transferase-mediated dUTP nick end-labeling
